# Image-guided Raman spectroscopy navigation system to improve transperineal prostate cancer detection. Part 2: *in-vivo* tumor-targeting using a classification model combining spectral and MRI-radiomics features

**DOI:** 10.1117/1.JBO.27.9.095004

**Published:** 2022-09-09

**Authors:** David Grajales, Fabien Picot, Roozbeh Shams, Frédérick Dallaire, Guillaume Sheehy, Stephanie Alley, Maroie Barkati, Guila Delouya, Jean-Francois Carrier, Mirela Birlea, Dominique Trudel, Frédéric Leblond, Cynthia Ménard, Samuel Kadoury

**Affiliations:** a Polytechnique Montréal, Montreal, Québec, Canada; b Centre de recherche du Centre Hospitalier de l’Université de Montréal, Montreal, Québec, Canada; c Institut du Cancer de Montréal, Montreal, Québec, Canada

**Keywords:** Raman spectroscopy, prostate cancer, tissue optics, multimodal imaging, machine learning, support vector machines, magnetic resonance imaging, ultrasound imaging

## Abstract

**Significance:**

The diagnosis and treatment of prostate cancer (PCa) are limited by a lack of intraoperative information to accurately target tumors with needles for biopsy and brachytherapy. An innovative image-guidance technique using optical devices could improve the diagnostic yield of biopsy and efficacy of radiotherapy.

**Aim:**

To evaluate the performance of multimodal PCa detection using biomolecular features from *in-situ* Raman spectroscopy (RS) combined with image-based (radiomics) features from multiparametric magnetic resonance images (mpMRI).

**Approach:**

In a prospective pilot clinical study, 18 patients were recruited and underwent high-dose-rate brachytherapy. Multimodality image fusion (preoperative mpMRI with intraoperative transrectal ultrasound) combined with electromagnetic tracking was used to navigate an RS needle in the prostate prior to brachytherapy. This resulting dataset consisted of Raman spectra and co-located radiomics features from mpMRI. Feature selection was performed with the constraint that no more than 10 features were retained overall from a combination of inelastic scattering spectra and radiomics. These features were used to train support vector machine classifiers for PCa detection based on leave-one-patient-out cross-validation.

**Results:**

RS along with biopsy samples were acquired from 47 sites along the insertion trajectory of the fiber-optics needle: 26 were confirmed as benign or grade group=1, and 21 as grade group >1, according to histopathological reports. The combination of the fingerprint region of the RS and radiomics showed an accuracy of 83% (sensitivity=81% and a specificity=85%), outperforming by more than 9% models trained with either spectroscopic or mpMRI data alone. An optimal number of features was identified between 6 and 8 features, which have good potential for discriminating grade group ≥1/grade group <1 (accuracy=87%) or grade group >1/grade group ≤1 (accuracy=91%).

**Conclusions:**

*In-situ* Raman spectroscopy combined with mpMRI radiomics features can lead to highly accurate PCa detection for improved *in-vivo* targeting of biopsy sample collection and radiotherapy seed placement.

## 1 Introduction

Transperineal biopsy and high-dose-rate (HDR) brachytherapy are two needle-based procedures for diagnosing and treating prostate cancer (PCa), respectively. HDR brachytherapy allows the delivery of a considerable dose of radiation to the tumor site using temporary implants while reducing the surrounding tissue involvement.^1^^,^^2^ Although using transrectal ultrasound (TRUS)-guided biopsy is the standard of care, it can have up to 30% false-negative rates and does not allow in situ characterization.^3^^,^^4^ It is clear that the efficiency of both techniques relies significantly on accurate localization of the tumor and needle.

Image-guidance is one of the strategies to support these localized procedures, using TRUS, which provides real-time anatomical information of the prostate and neighbor structures. It, therefore, provides navigation support for biopsies or brachytherapy catheter implantation, but since not all lesions are hypoechoic, it does not provide information on tumor location.^5^^,^^6^ On the other hand, magnetic resonance imaging (MRI), more specifically multiparametric MRI (mpMRI) sequences based on diffusion, offer higher sensitivity, allowing the visualization of certain lesions. Currently, mpMRI is used by physicians to report tumors (PIRADSv2.1^5^^,^^7^) and plan interventions, but it also allows the extraction of quantitative features (radiomics), which could be used as biomarkers.^2^^,^^8^^,^^9^ However, MRI presents certain limitations to guide interventions due to longer acquisition time, limited compatibility with surgical instruments, and cost.^1^^,^^10^ Multimodal image registration is currently studied and used to take advantage of complementary information of TRUS and MRI to assist HDR brachytherapy and other tumor-targeted prostate interventions.^10^^–^^13^

This approach, combined with electromagnetic (EM) tracking, helps provide navigation capabilities for targeting tumors.^14^^–^^16^ However, neither of these modalities allows *in-situ*, real-time tissue characterization, which could significantly impact the diagnosis and treatment efficacy, reducing false-negative rates and boosting personalized treatments for more than 1.4 million PCa new cases diagnosed every year worldwide.^17^^–^^20^

Raman spectroscopy (RS), on the other hand, characterizes microscopic information of prostate tissue, providing real-time molecular signatures and taking advantage of the tissue’s highly sensitive and specific optical properties.^18^^,^^21^^–^^24^ Based on inelastic light scattering, RS has been used for years for *ex-vivo* sample characterization, producing spectra with molecular vibrational states information, showing great potential for detecting several diseases.^25^^–^^28^ Furthermore, with the development of optical fiber RS probes, this technique is moving to clinical applications^29^; different optical probe designs have been used for *in-vivo* tissue characterization (in human and animal models) for targeting skin cancer in open surgeries,^30^ minimally invasive diagnosis of lung cancers,^31^ bladder cancer detection using a superficial and nonsuperficial Raman probes,^32^ observation of skin changes after breast cancer treatment,^33^ and others.^34^^–^^37^ In prostate applications, it has been used for *ex-vivo* characterization and *in-vivo* margin detection,^19^^,^^20^^,^^23^^,^^38^^,^^39^ but, to the best of our knowledge, so far, not for real-time *in-vivo* prostate tumor burden confirmation, which can provide great benefit for clinical procedures.

As previously described, mpMRI has remarkable tumor-related information on a larger scale, especially for diffusion-weighted sequences, where radiomics can extract this information quantitatively.^8^^,^^13^^,^^40^ There is a wide variety of standardized radiomics features primarily classified as intensity-, texture-, or shape-based. Shape-based are especially useful when lesions are segmented; the other two classes have been studied, identifying potential on some first order and some gray-level-correlation-matrix (GLCM) features.^41^^,^^42^

Multimodal and multiscale characterization is advantageous for tissue characterization purposes given the complementary information it provides, as single modalities may not capture all critical elements of the interrogated sample.^24^^,^^38^^,^^43^^,^^44^

This pilot clinical study aims to evaluate the feasibility of a multimodal and multiscale characterization approach for *in-vivo* PCa classification during clinical procedures. We combined real-time mesoscopic characterization provided by RS and macroscopic characterization from preoperative mpMRI, co-localized with multimodal image registration and EM tracking, as input for a support vector machine (SVM), for assessing the classification potential of such characterization. This paper is the second part of a joint work carried out in the framework of the same pilot clinical study; part 1^45^ presents details of the optical system, results of *ex-vivo* experiments, and their comparison with *in-vivo* results.

## 2 Materials and Methods

### 2.1 Clinical Data and Equipment

This pilot clinical study was conducted between September 2020 and August 2021, with 18 patients with histological diagnosis of PCa, enrolled on a prospective clinical trial approved by the Research Ethics Board (NCT03378856).

Planning mpMRI (3D T2-weighted FSE, b2000 DWI, +/−DCE) were obtained on a 1.5T Siemens Aera Magnetom (Siemens Healthineers, Erlangen, Germany) using surface coils. Voxels on acquired T2 images were $1\times 1\times 1\;{\mathrm{mm}}^{3}$, b2000 images (diffusion-weighted images b-value of $2000\;{s/\mathrm{mm}}^{2}$) consisted of $2.6\times 2.6\times 5\;{\mathrm{mm}}^{3}$ voxels, and voxels on calculated ADC maps were $1.8\times 1.8\times 4\;{\mathrm{mm}}^{3}$. Positron emission tomography/computed tomography (targeting prostate-specific membrane antigen: PSMA-PET/CT, 18F-DFCPyL)^22^ images were also acquired in a subset. For intraoperative imaging, a bk3000 ultrasound system was used with a BK endocavity biplane transducer (BK Ultrasound, Herlev, Denmark). Brachytherapy procedures were assisted by a prototype interventional system (Invivo/UroNav, Philips Disease Management Solutions, Gainesville) in the early phase of clinical deployment.

**Fig. 1:**
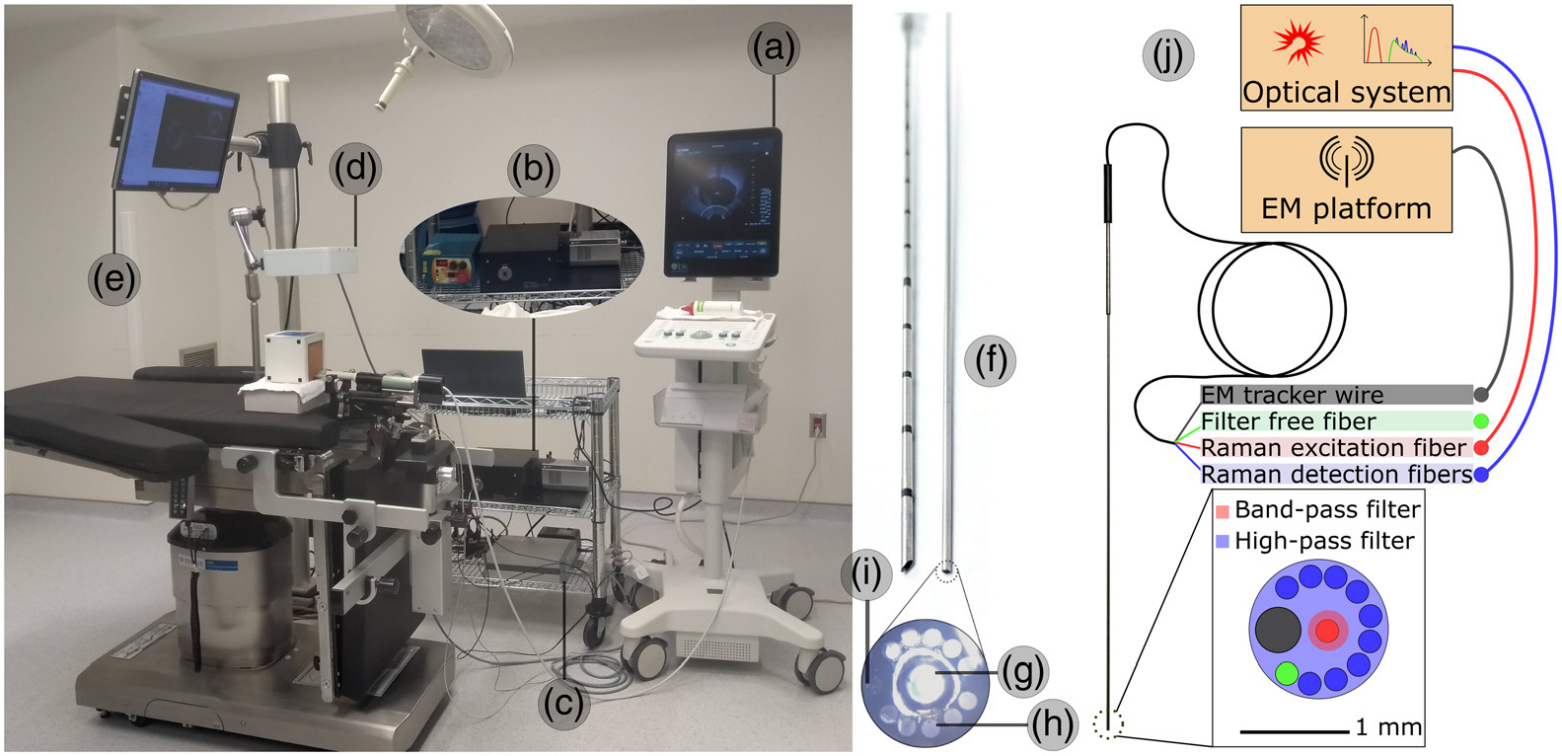
Clinical setup: (a) ultrasound system; (b) near-infrared laser and spectrometer; (c) EM tracking system; (d) EM field generator; (e) 3D Slicer navigation system; (f) closeup of the RS probe fiber bundle next to cannula; (g) Raman excitation fiber; (h) Raman detection fibers; (i) EM sensor; and (k) schematic illustration of optical setup.^20^^,^^45^

We used a custom system consisting of navigation and optical components (Fig. 1). The optical component contains a dual source (671 and 785 nm, Semrock, New York), a spectrometer (EmVision LLC, Fl), and the custom EM tracked optical probe (EmVision LLC, Fl), designed to perform *in-situ*, minimally invasive characterization.^45^ The use of this subsystem, controlled by customized MATLAB R2017b (Mathworks, Massachusetts) software, allows one to stimulate the tissue and detect energetical shifts due to inelastic scattering from light–tissue interactions, which is correlated to molecular vibration modes.^19^

The navigation component is connected to the ultrasound system and uses MRI-TRUS fusion to project structures segmented on MRI over the real-time TRUS images. It is also connected to the EM tracking platform (Aurora NDI, Waterloo, Canada) consisting of a control unit, an EM field generator placed over the patient’s pelvic region, and three six-degrees-of-freedom EM sensors: the first one placed on the template as a fix reference, the second fixed to the TRUS-probe holder to track the field of view of the current 2D image, and the third one, small enough to be integrated to the custom optical probe lumen, to reconstruct the probe while navigating to pre-identified targets. A 3D Slicer module was created for visualization and control of the subsystems.^46^ More details on the system could be found in previous works.^20^^,^^47^

### 2.2 Workflow and Data Acquisition

HDR brachytherapy procedures include the following steps: (i) importing the preoperative images and predetermined contours into the interventional system, (ii) performing a 3D reconstruction from 2D TRUS image and segmenting the prostate, (iii) registering images and propagating the MRI contours to TRUS, (iv) inserting and reconstructing the catheters, and calculating the dose plan, and finally, (v) delivering the dose. All these steps were performed with the patient under general anesthesia and in lithotomy position. Details of the intervention (brachytherapy workflow), image registration, and EM tracking can be found in Ref. 48.

**Fig. 2:**
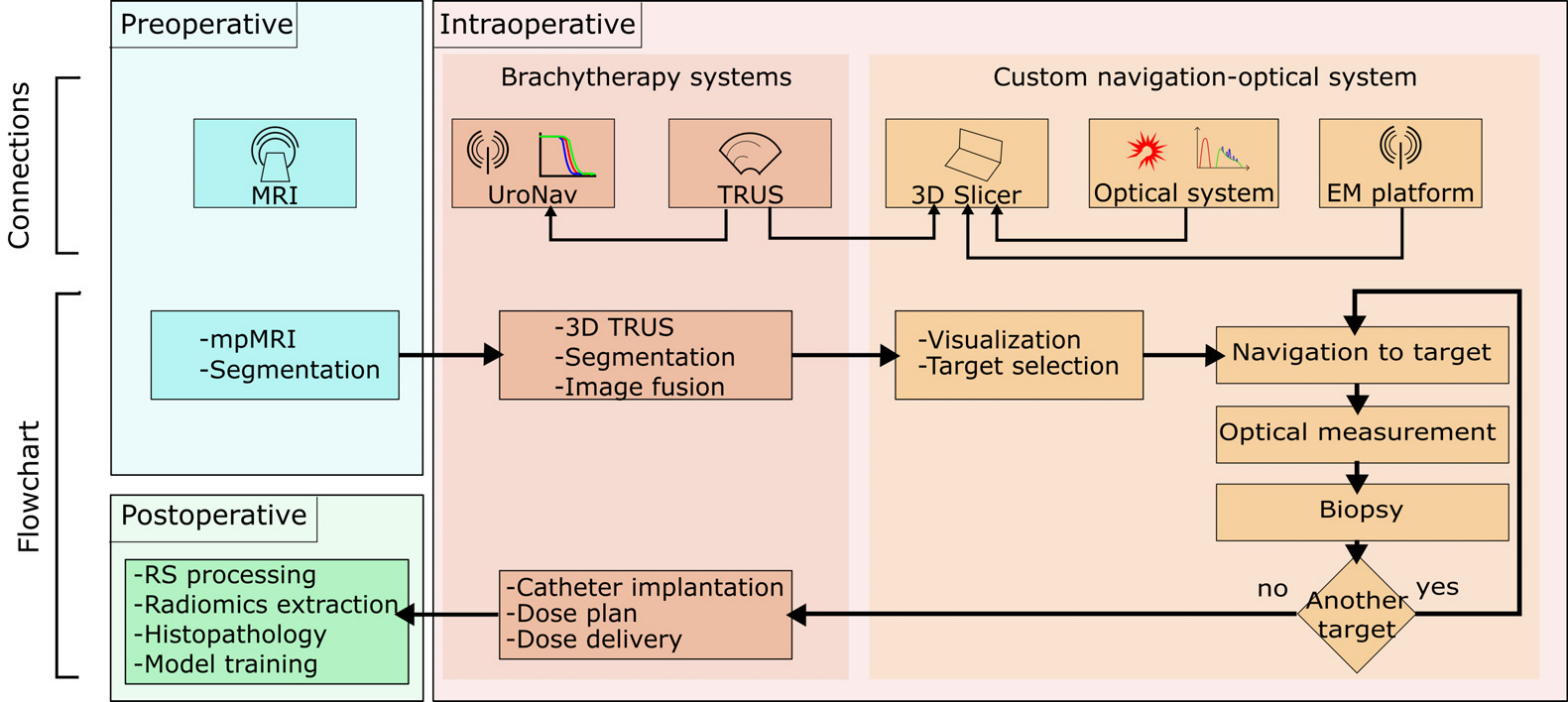
Flowchart and connections diagram of the systems involved in the intervention. The larger boxes divide the pre-, intra-, and post-operative phases of the procedure. The left column in the intraoperative section contains the equipment and activities inherent to brachytherapy. The right column contains the steps and systems added to the interventions to carry out the present study.

The optical acquisitions were performed during HDR brachytherapy procedures after the elastic image registration (defined below); once the optical measurements were completed, the brachytherapy procedure resumed. The general diagram of the connections and the flow diagram can be seen in Fig. 2.

Gross tumor volumes (GTV) were segmented on T2 MRI, based on mpMRI and PSMA-PET images; prostate and urethra were segmented in preoperative images (using Eclipse, Varian Medical Systems, Palo Alto) and intraoperative images (using the interventional system). Additionally, T2 images and contours were elastically registered to the intraoperative TRUS, using a surface-based algorithm integrated into the prototype interventional system; for this process, the prostate contours (from MRI and TRUS) were automatically centered, then manually aligned following the urethra angle, and then automatically deformed looking for an optimal surface correspondence. TRUS images and propagated contours were exported to our custom navigation system, enabling visualization of deformed structures (e.g., GTV) projected over real-time TRUS.

Targets (e.g., GTV center of mass, healthy tissue far from the GTV) were pre-identified based on the preoperative MRI, and the optical probe was navigated to them supported by a coaxial needle (cannula). Once at the site of interest, the dual optical source stimulated the tissue, and the spectrometer captured the response signal (from 50 to 100 RS spectra per site). The coordinates of the inspected sites in the TRUS reference system were recorded, and a confirmation biopsy was taken at the same location.

Biopsy cores were fixed and processed according to standard histopathologic procedures observed by an expert to identify patterns on the stained sample slides.^49^ According to the predominant and secondary patterns, a report was generated presenting the Gleason score (GS), the grade group according to the International Society of Urological Pathology (ISUP GG), and the high-grade tumor percentage (HG) for each biopsy core.^50^ For instance, a slide presenting tumor tissue, consisting of 80% pattern 4 and 20% pattern 3, will be reported as GS: 4+3=7, ISUP GG: 3/5, and HG:80%.^51^ Due to the impact over treatment planning, classification algorithms for PCa are usually trained for detecting tumors with ISUP  GG>1, so initially, we set our “ISUP  GG>1” prediction task by labeling the observations with ISUP  GG≤1 (including ISUP  GG=1 and benign tissue) as false and sites with ISUP  GG>1 as true.^1^^,^^41^^,^^52^

**Fig. 3:**
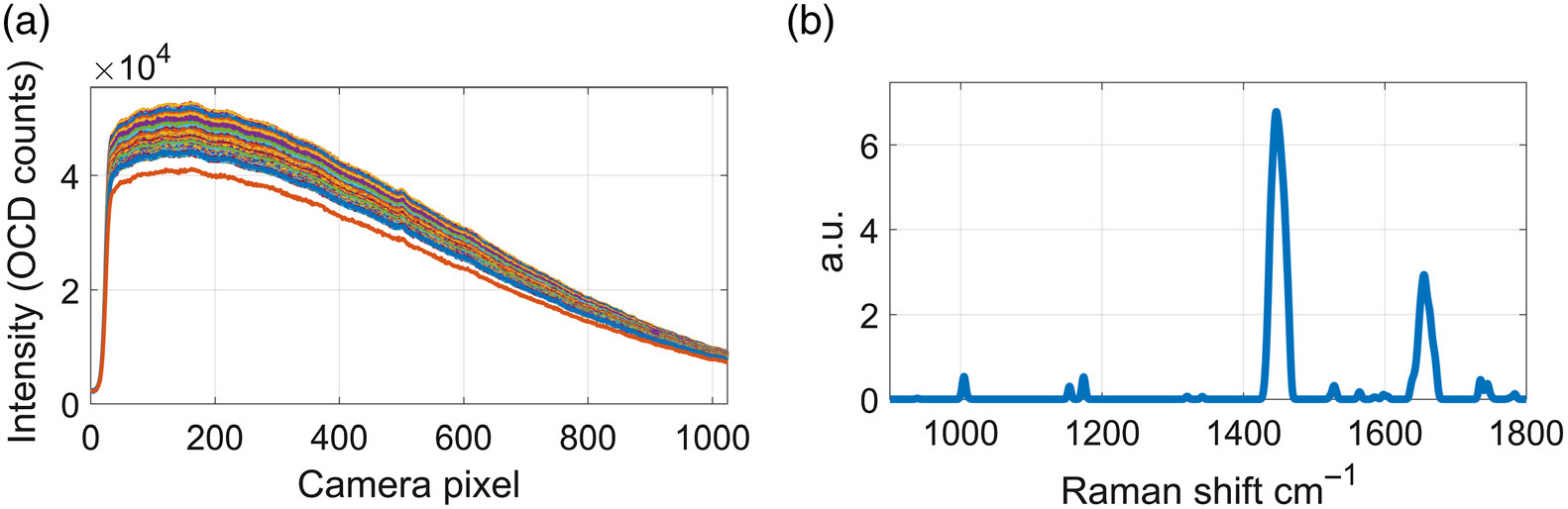
Sample signal processing. (a) About 50 raw fingerprint acquisitions from one inspected site and (b) the resulting processed Raman spectrum.

The RS signal processing consisted of averaging all the spectra from one site, removing autofluorescence and cosmic rays, standard normal variate normalization, and finally assigning each pixel of the spectrometer to a Raman shift (Fig. 3).^45^ We obtained a single spectrum per site at the end of this process. This was applied for the fingerprint (FP) and the high wavenumber (HW) region of the RS, according to the source used to stimulate the tissue. For this study, every single Raman shift on the spectrum was a feature for the next steps.^47^

To extract MRI-based features (Rad), the PyRadiomics platform was used,^42^ which offers up to 120 features from different categories; based on literature, we selected 8 first order and 8 GLCM features (Table 1) to be calculated on T2, ADC, and b2000 mpMRI.^8^^,^^40^^,^^41^^,^^52^ Using the transformation matrix employed to register MRI and TRUS, we applied the inverse process to find the corresponding coordinates on the mpMRI for each inspected site. We calculated the radiomics features in a 5-mm radius spherical volume at each position.

**Table 1:** Radiomics features extracted from the three mpMRI sequences, and their identifiers.

Group	Name	Identifier		
		T2	ADC	b2000
First order	Energy	r1	r17	r33
	Total energy	r2	r18	r34
	Entropy	r3	r19	r35
	Mean	r4	r20	r36
	Median	r5	r21	r37
	Standard deviation	r6	r22	r38
	Mean absolute deviation	r7	r23	r39
	Uniformity	r8	r24	r40
GLCM	Auto correlation	r9	r25	r41
	Cluster shade	r10	r26	r42
	Contrast	r11	r27	r43
	Correlation	r12	r28	r44
	Id (inverse difference)	r13	r29	r45
	Difference entropy	r14	r30	r46
	Joint entropy	r15	r31	r47
	Joint energy	r16	r32	r48

### 2.3 Classification Model Training

Given the low number of patients, which is inherent to a pilot clinical study, we decided to limit the complexity of the classification model as a strategy to avoid overfitting issues. Thus, we trained SVM models for binary classification, with a linear kernel and a cost matrix that doubles the penalty to false negatives (C=[0,1;2,0]), using MATLAB R2017b. For training and validation, we followed a leave-one-patient-out cross-validation (LOPOCV) scheme (i.e., models were trained with data from all patients but one, which was used for validation).

In a high-dimensional dataset, especially with a limited number of observations and patients, feature selection is crucial. To perform this step, we set a maximum number of features to be selected (max_nf) and applied a three-step selection approach:

i. Amount of variation: the principle is to discard the features that have almost the same value for all the observations. We calculated the variance for each feature and only retained the features with var(X)>0.03 for the next selection step.
ii. Correlation with the target: we calculated the correlation coefficients between each feature and the assigned label (ground truth), then discarded the features with an absolute value of correlation <10%.
iii. Lasso regression: this method assigns a weight to each feature within an optimization function and gives a non-zero weight only to features that contribute significantly to establishing a decision boundary, which means that the number of selected features could be equal to or less than the maximum number of non-zero coefficients we set (max_nf).

This three-step process was applied after dividing training/validation sets, i.e., the observations of the patient left out were not considered during the feature selection. As a result, the number of features selected for each fold may vary slightly, so the value reported in the results is the average of all folds.

We describe below three sets of experiments focused on the classification potential of collected features (RS and Rad) to train the predictive model.

#### 2.3.1 Feature combination experiment

We trained different models using independent sets of features (FP, HW, or Rad), as well as the different combinations between them (FP+HW, FP+Rad, FP+HW+Rad, and HW+Rad). For this experiment, we arbitrarily set a max_nf=10, trying to limit the number of features, close to 50% of the number of patients. Using the posterior probability for each measurement used for validation, we plotted the receiver operating characteristic (ROC) curve for each model to describe the general performance comparing the area under the curve (AUC). We then calculated the accuracy, sensitivity, and specificity to evaluate the performance at the optimal threshold.

#### 2.3.2 Number of features experiment

Using the combination of features demonstrating the best performance in the previous experiment (Sec. 2.3.1), we trained a model increasing the max_nf  value for the selection process (from 2 to 18) to find the optimal number of features for the classification task. Then, we identified the features that, among the 18 iterations of the LOPOCV, were selected more than 10 times (most frequently selected features).

#### 2.3.3 Prediction task experiment

The experiments presented in Secs. 2.3.1 and 2.3.2 were focused on ISUP  GG>1 prediction using the labels we described in Sec. 2.2. For this experiment, we applied different criteria to the histopathological results (the limit for class selection) to assign labels for two other prediction tasks. For the first one, we labeled the observations with ISUP  GG≥1 as true and benign tissue as false (ISUP  GG≥1 prediction). For the second one, “high grade” prediction, we used the same labels from the previous one but excluded the observations with an HG<20%. We used the most frequently selected features identified in the previous experiment (Sec. 2.3.2) to train the models, with no other feature selection.

## 3 Results

### 3.1 Clinical Data

**Fig. 4:**
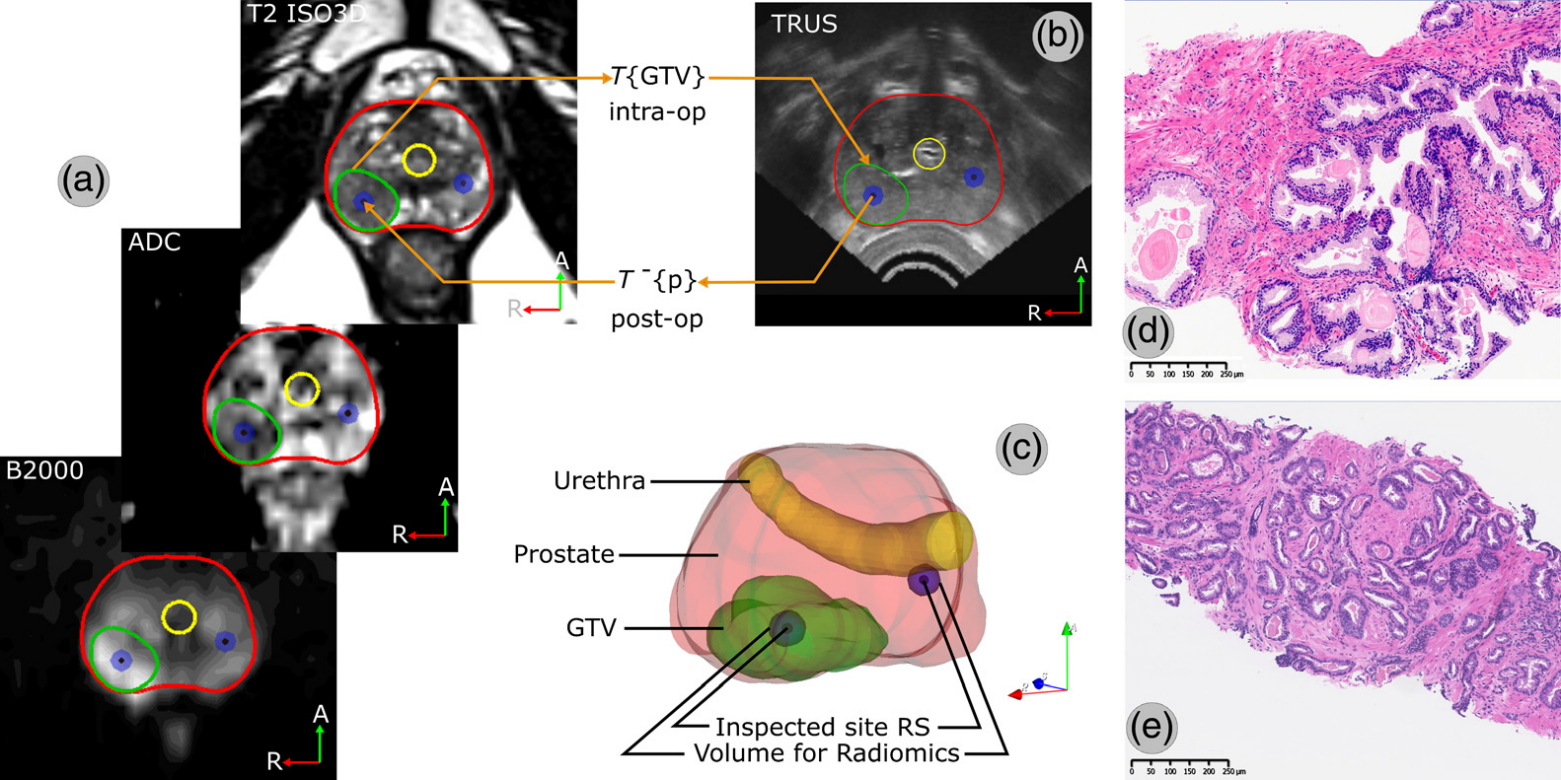
Sample image registration and co-location results. (a) GTV originally segmented on preoperative mpMRI is (b) projected over the real-time TRUS using a surface-based elastic registration algorithm; the inverse process allows identifying the MRI coordinates corresponding to the inspected sites. (c) A 3D model of the plan can be rendered. (d) Pathological image samples of benign prostatic parenchyma and (e) acinar adenocarcinoma GS: 3+4=7, ISUP GG=2, and HG: 40% to 50%.

In total, the dataset consisted of 47 inspected sites, with the corresponding histopathological report for ground truth, and 4650 features (Table 2). Sample images of two histopathological slides are presented in Fig. 4.

**Table 2:** Clinical data.

Number of patients	18
Median age (years)	68 (range: 60–74)
Median inspected sites/patient	2 (range: 2–5)
Total inspected sites	47
Benign	23
GS: 3+3=6/ISUP GG: 1/5	3 (3/3)^a^
GS: 3+4=7/ISUP GG: 2/5	10 (3/10)^a^
GS: 4+3=7/ISUP GG: 3/5	8
GS: 4+4=8/ISUP GG: 4/5	3
Total number of features	4650
FP (RS)	1801
HW (RS)	2801
Rad	48
Mean added time (min)	18.4 (SD=4.9)

a Number of samples with HG<20%.

Elastic registration allowed the projection of the deformed GTV contours on real-time TRUS, which was used to set up to five sites of interest and for navigation support during the intervention; the inverse process allowed the co-location of the inspected sites on the mpMRI, post-intervention [Figs. 4(a)–4(b)]. According to the voxel size of mpMRI and the volume defined for radiomics extraction (blue sphere on Fig. 4), ranges of 428–482, 34–38, and 24–32 voxels were used from T2, ADC, and b2000, respectively.

### 3.2 Feature Combination Experiment

**Fig. 5:**
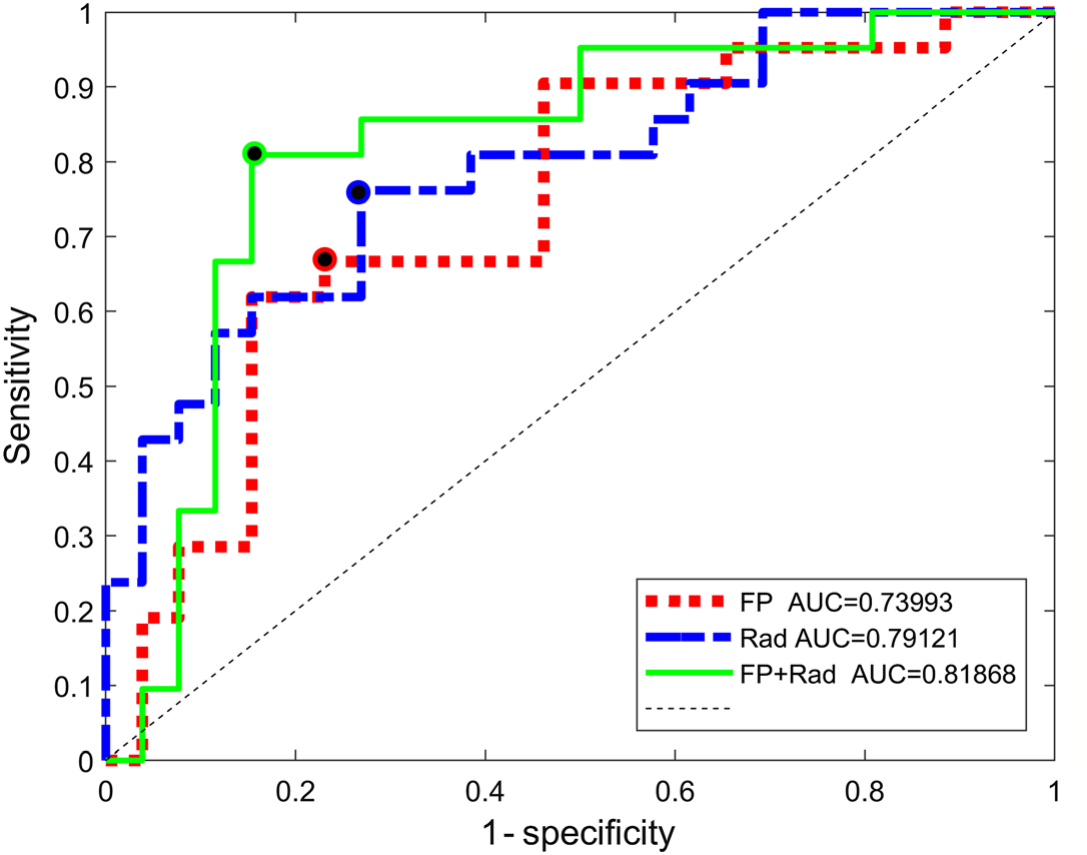
ROC curve and optimal point for discriminating ISUP GG>1 and ISUP GG≤1, for models including feature selection (max_nf=10). AUC: Area under the curve.

Following a dichotomization of the collected data during the brachytherapy procedures (ISUP  GG>1 criteria), these experiments consisted of 21  ISUP  GG>1 and 26  ISUP  GG≤1 samples. The AUC results for the different combinations of features are shown in Table 3, and the ROC curves for the groups of features involved in the model with the best performance are presented in Fig. 5.

**Table 3:** Classification performance of SVM models (LOPOCV) for discriminating ISUP GG>1 and ISUP GG≤1.

Features	Number features	AUC	Prediction accuracy	Sensitivity	Specificity	SV rate
FP	8.3	0.74	0.72	0.67	0.77	0.64
HW	8.3	0.55	0.53	0.48	0.58	0.76
Rad	8.4	0.79	0.74	0.76	0.73	0.70
FP+HW	8.2	0.80	0.70	0.78	0.64	0.62
FP+Rad	8.3	0.82	0.83	0.81	0.85	0.55
FP+HW+Rad	8.2	0.79	0.81	0.71	0.88	0.52
HW+Rad	8.6	0.58	0.62	0.67	0.58	0.64

Note: SV rate: number of support vectors/total of observations.

The FP+Rad model showed the best overall performance, based on AUC, followed closely by three other combinations: FP+HW, rad, and FP+HW+rad. From these four models, FP+Rad and FP+HW+Rad yielded a low mean support vectors ratio (number of support vectors needed to train the model, divided by the total number of observations) compared to the other two models.

Prediction accuracy, sensitivity, and specificity were calculated at the optimal point on the ROC curve, which is the closest point to the (0,1) corner of the plot. These metrics also showed the superior performance of FP+Rad (Table 3). Regarding the prediction accuracy, FP+Rad outperformed all other models but one for more than 9%; FP+HW+Rad, which also combined optical and image-based features, was the only model close to this accuracy (only 2% below) but also 10% below in sensitivity.

### 3.3 Number of Features Experiment

**Fig. 6:**
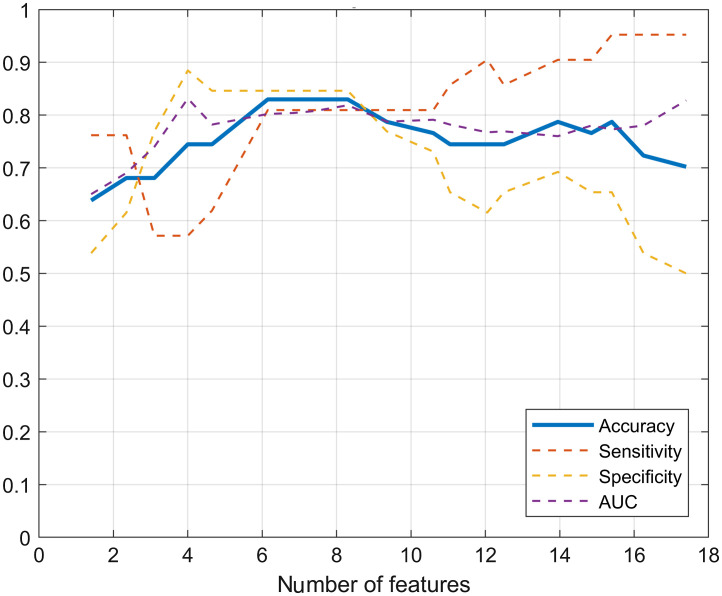
Classification performance of the FP+Rad model in function of the number of features selected.

Using the combination of features with the best overall performance (FP+Rad) identified in the previous experiment (Sec. 3.2), we trained models varying max_nf for the feature selection process. As explained in Sec. 2.3, the selected features for each fold (LOPOCV) could vary slightly, up to the set limit (max_nf), so the actual number of features reported is the mean number of features selected for all the folds. According to AUC results and metrics calculated at the optimal point (Fig. 6), there is an optimal region between 6.1 and 8.3 used features, resulting when setting the max_nf at 7 and 10, respectively. Limiting the number of features used to train the classification model aimed to reduce overfitting given the small datasets and improve generalization capabilities; but the remarkable drop in specificity observed in the graph for more than nine features emphasizes the importance of the feature selection process.

**Fig. 7:**
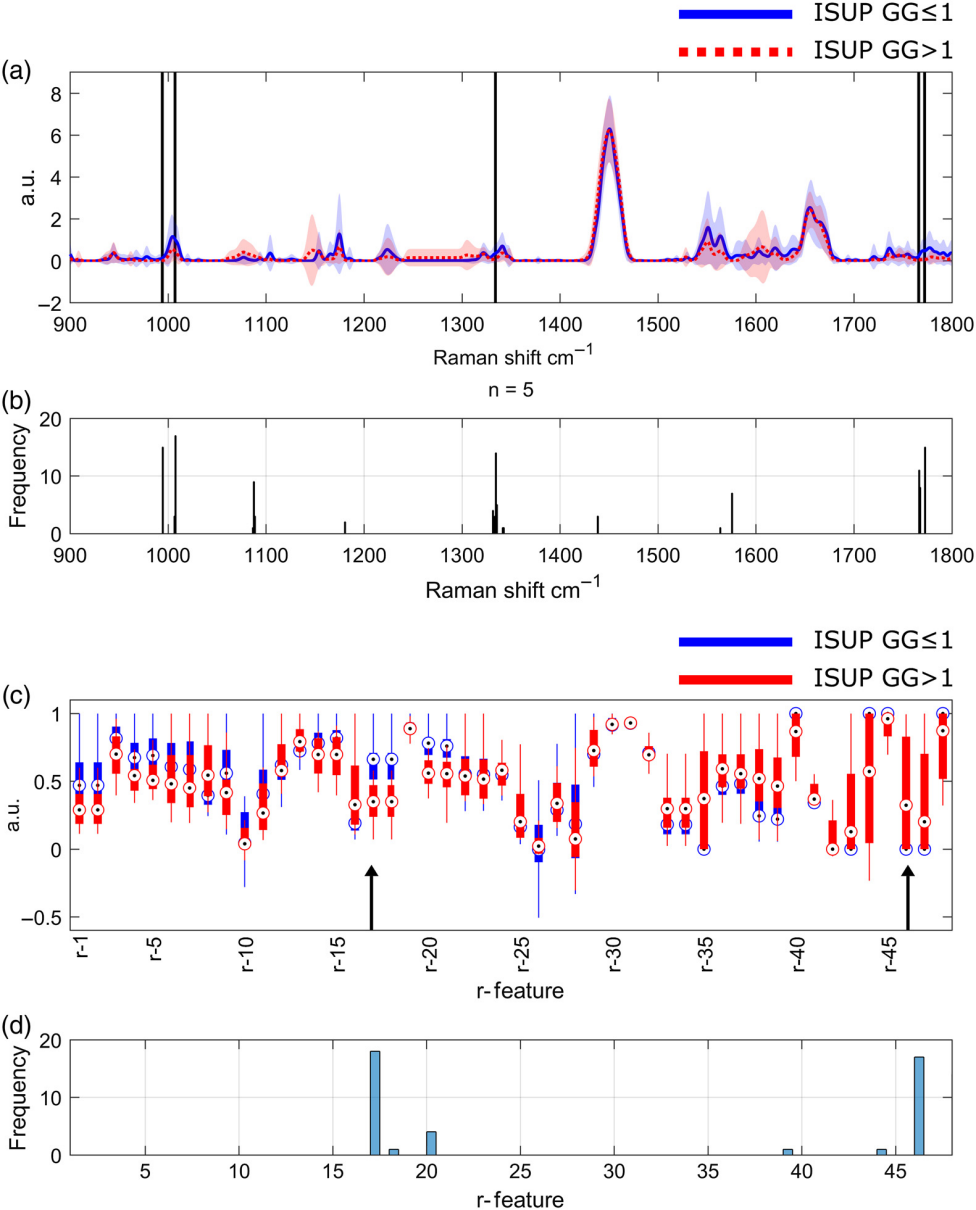
Average and standard deviation (a) of processed FP Raman spectra and (b) histograms of selected features for RS; average and standard deviation (c) of mpMRI radiomics and (b) histograms of selected radiomics features. The radiomics features corresponding to each identifier are given in Table 1.

The features selected on each fold for the model that used on average 8.3 features were not always the same, so Figs. 7(b)–7(d) shows how many times (# of folds) each feature was selected. FP Raman spectra were averaged by class for visualization [Fig. 7(a)], and from those 1801 features (Raman shifts), five features were selected more than 10 times (i.e., during the feature selection applied over each fold): 994, 1007, 1334, 1766, and $1772\;{\mathrm{cm}}^{-1}$. Similarly, radiomics features were averaged by class [Fig. 7(c)], and two features were selected more than 10 times: r17 (Energy from ADC) and *r*46 (DifferenceEntropy from B2000), both selected on all the folds (18 times). This total of seven most frequently selected features could make a set of features with great potential for further studies.

It can be observed, as expected, that the valleys in the Raman spectrum were not selected, except for some features just at the base of a significant peak, which contained information on the width of the peak (e.g., 1108 or $1575\;{\mathrm{cm}}^{-1}$). As for the radiomics features, it can be seen that, apart from the two most frequently selected features, only a few were selected at least once (r18, r20, r39, and r44); according to the selection method, none of the T2 features, GLCM from ADC, and first order from b2000 contributed to the classification task.

### 3.4 Prediction Task Experiment

We can observe that the different criteria to set the classes for the prediction tasks resulted in different numbers of labels and observations (Table 4). The three observations with ISUP GG=1 (Table 2) were HG<20%, so the last set of labels (high grade) did not include these samples.

**Table 4:** Classification performance using the most frequently selected features, based on different prediction tasks.

Prediction task	ISUP GG>1	ISUP GG≥1	High grade
Total inspected sites	47	47	41
True class (+1)	21 ISUP GG>1	24 ISUP GG≥1	18 ISUP GG≥1, HG>20%
False class (−1)	26 ISUP GG≤1	23 ISUP GG<1	23 ISUP GG<1
Accuracy	0.91	0.87	0.88
Sensitivity	0.90	0.83	0.89
Specificity	0.92	0.91	0.87

**Fig. 8:**
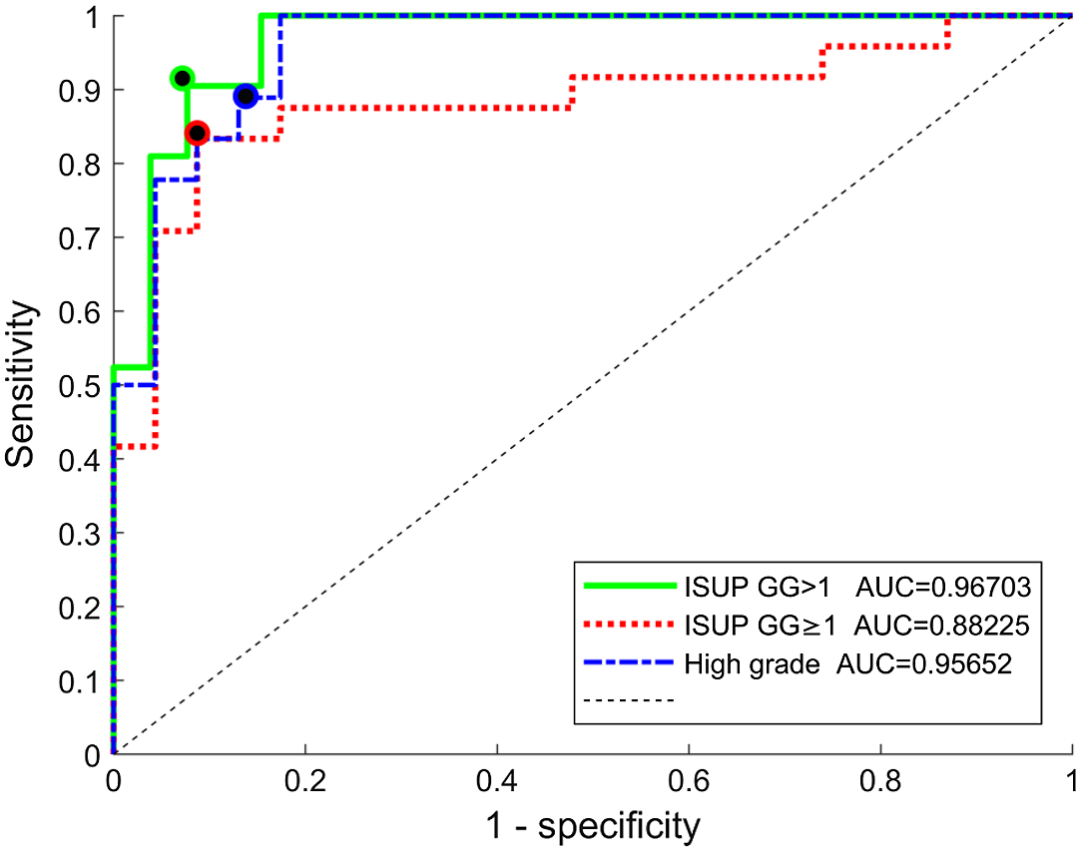
ROC curve and optimal point for models trained with the seven most frequently selected features, for the three different prediction tasks (ISUPGG>1, ISUPGG≥1, and high grade).

The models trained for each prediction task were compared in terms of general performance (ROC) and the performance at the optima point, and the results are shown in Fig. 8 and Table 4.

## 4 Discussion

This study evaluated the potential of using macroscopic and mesoscopic tissue characterization for PCa classification purposes, combining *in-vivo* RS and radiomics from preoperative mpMRI, for detection of ISUP GG>1 assisted by a custom navigation system. As in different commercial devices for other applications, our navigation system is able to guide the physician during the insertion of the optical probe and track it in real-time in correspondence to pre-operative MRI, which contributes to a time-efficient procedure, by adding an acceptable time to the procedure.

Previous studies showed the important potential of RS for PCa detection; for instance, *ex-vivo* studies^20^^,^^39^^,^^53^ presented a classification accuracy ranging between 83% and 89%. For some of these studies, in addition to not being carried out under *in-vivo* conditions, models were trained with at least 10 times more *ex-vivo* data; this prepares the model to overcome data variability and widens the range of features to be used (no feature selection in some cases). These differences (*ex-vivo*/ *in-vivo*, size of the dataset, and number of features) may be the reason why the RS models alone are not as performant, especially using HW; however, it should be noted that FP and FP+HW have accuracy larger or equal to 70% using just 10 features for *in-vivo* conditions.

There are models trained only with MRI-based features that achieve AUC above 80% in the literature.^41^^,^^52^ For instance, a model trained with nine radiomics features achieved a sensitivity of 84% and a specificity of 73%, detecting clinically significant PCa better than the Rad model.^52^ Nevertheless, they used some shape-based features that need the tumor’s segmentation and some features extracted after using different filters on the images. The relatively simpler features used in this study simplify the processing of the images, do not require the segmentation of the GTV, and allow the extraction of the features at the place where the probe is placed; this is essential for potential real-time applications.

As multimodal information provides significant advantages for navigation, different characterization modalities are also advantageous for classification purposes.^43^ One of the models that combined optical and image-based properties exhibited the best performance: FP+Rad. The complementary information of mesoscopic and macroscopic characterization allowed FP+Rad to outperform the models that only used a single modality (FP or Rad) on all metrics. Data variability is difficult to model in small datasets; features coming from different modalities, principles, or equipment could contribute to model generalization. The performance of this model, using eight features from two different modalities, is comparable to the studies mentioned before that use more features from a single modality in *ex-vivo* conditions.

The combination of FP + Rad was the only model that yielded a sensitivity and specificity over 80%, which is very important since prostate-specific antigen is known for its high false positives rates, and TRUS-guided biopsies can lead to a high number of false negatives. Some mpMRI- or PET-based methods have greater sensitivity and specificity (respectively) for the localized characterization of PCa, but require time-consuming processing by experts or could have potential side effects for some tracers.^41^^,^^48^^,^^54^

The most frequently selected Raman shifts were related to three important spectra bands that are usually more predominant in healthy (or ISUP  GG≤1) tissue. The first one, from 990 to $1015\;{\mathrm{cm}}^{-1}$, has a central peak usually related to proteins (phenylalanine).^22^^,^^39^^,^^55^^,^^56^ From 1330 to $1350\;{\mathrm{cm}}^{-1}$, the second band is commonly associated with collagen, DNA, or RNA.^22^^,^^56^ Finally, the band from 1760 to $1790\;{\mathrm{cm}}^{-1}$, influenced by DNA/RNA, proteins and phospholipids, is also present on benign prostatic hyperplasia.^23^^,^^56^

Energy, as a radiomics feature, is the measure of the magnitude of the value (square intensity of segmented voxels), so the lower the intensity is on the image, the lower metric. Low intensity in ADC images is generally associated with tumors, which is consistent with the higher mean value for ISUP  GG≤1 tissue for this feature calculated on ADC images (r17).^40^^,^^57^

The loss in performance when using only the samples with HG >20% instead of using all the acquisitions (high grade versus ISUP  GG>1) was very limited (∼1% on AUC and 3% on accuracy). This indicates that, despite having limited datasets, by eliminating 6 observations (and using the same features), the model still performed adequately, which can be explained by the pooling of features from macro- and mesoscopic levels and the fact that the model uses less than 60% of the observations as support vectors. Comparing the ISUP  GG>1 versus ISUP  GG≥1 prediction tasks, the performance at the optimal point is similar (∼4% difference in accuracy), projecting the potential of the approach for both classification tasks. The slightly better performance in this experiment (prediction task experiment) compared to the initial one (feature combination experiment) may stem from the fact that the seven features were chosen based on the selection of all folds, not on our LOPOCV scheme.

This pilot clinical study has some limitations, such as the limited number of patients enrolled, making the feature selection process critical. The selection approach was useful to exclude many silent features, but also to choose a small number that is comparable, ideally smaller, than the number of observations. Other limitations are related to some elements that may compromise the correct co-localization of the optical acquisitions and histopathological results; although the navigation system helped and the performance for high grade was correct (when removing the samples that could be less certain), the co-location of the ground truth would be an aspect to improve for the following stages.

The implemented approach has facilitating elements for performing a minimally invasive classification in real-time. However, for future work, the signal/image processing and the pre-trained classification model need to be integrated into the navigation system to get a complete system for *in-vivo* real-time studies.

## 5 Conclusion

We demonstrated that complementary information from *in-situ* RS and mpMRI radiomics features allowed to accurately stratify the ISUP  GG  >1/ISUP  GG  ≤1, as well as discriminate ISUP  GG≥1/ISUP  GG  <1 sites using SVM classifiers. This classification performance, combined with our custom navigation system, can lead to an accurate PCa detection and localization, improving tumor targeting in minimally invasive interventions.
